# Development of a Light-Weight Unmanned Aerial Vehicle for Precision Agriculture

**DOI:** 10.3390/s21134417

**Published:** 2021-06-28

**Authors:** Uchechi F. Ukaegbu, Lagouge K. Tartibu, Modestus O. Okwu, Isaac O. Olayode

**Affiliations:** Department of Mechanical and Industrial Engineering, University of Johannesburg, Johannesburg P.O. Box 2028, South Africa; ltartibu@uj.ac.za (L.K.T.); okwu.okechukwu@fupre.edu.ng (M.O.O.); olayode89@gmail.com (I.O.O.)

**Keywords:** unmanned aerial vehicle (UAV), deep learning, Raspberry Pi 3, industry 4.0, precision agriculture

## Abstract

This paper describes the development of a modular unmanned aerial vehicle for the detection and eradication of weeds on farmland. Precision agriculture entails solving the problem of poor agricultural yield due to competition for nutrients by weeds and provides a faster approach to eliminating the problematic weeds using emerging technologies. This research has addressed the aforementioned problem. A quadcopter was built, and components were assembled with light-weight materials. The system consists of the electric motor, electronic speed controller, propellers, frame, lithium polymer (li-po) battery, flight controller, a global positioning system (GPS), and receiver. A sprayer module which consists of a relay, Raspberry Pi 3, spray pump, 12 V DC source, water hose, and the tank was built. It operated in such a way that when a weed is detected based on the deep learning algorithms deployed on the Raspberry Pi, general purpose input/output (GPIO) 17 or GPIO 18 (of the Raspberry Pi) were activated to supply 3.3 V, which turned on a DC relay to spray herbicides accordingly. The sprayer module was mounted on the quadcopter and from the test-running operation conducted, broadleaf and grass weeds were accurately detected and the spraying of herbicides according to the weed type occurred in less than a second.

## 1. Introduction

Farmers are often faced with the challenges of eradicating weeds on farmland. Weeds are unwanted plants which compete with crops for water, light, nutrient, and space on farmland. This struggle has adverse effects on crop yield, growth, and development. Unfortunately, local farmers in developing countries often use the traditional weed control methods, which involve manual removal of weeds and manual application of herbicides on a large agricultural field, which is laborious, time-wasting, and often leads to environmental pollution. In this era of the fourth industrial revolution, sophisticated technologies which involve the use of flying and ground robots in spraying herbicides, have become necessary. Hence, this research focuses on proffering solutions aligned with Industry 4.0 to address this prevalent problem and its resultant consequences. To increase food production by 2050 and feed a population of about nine billion, the concept of precision agriculture through the application of technological solutions is imperative. Precision or smart agriculture involves optimizing agricultural processes, real-time analysis, and monitoring of agricultural data [1]. It also involves automation, making intelligent predictions based on algorithms and various approaches aimed to maximize economic returns while preserving resources and protecting the environment [2]. According to Miranda et al. [3], the word “smart” relates to the ability of a system to integrate control and actuation tasks in analyzing situations to make real-time decisions given available data.

### 1.1. Unmanned Aerial Vehicles (UAV)

**Figure 1:**
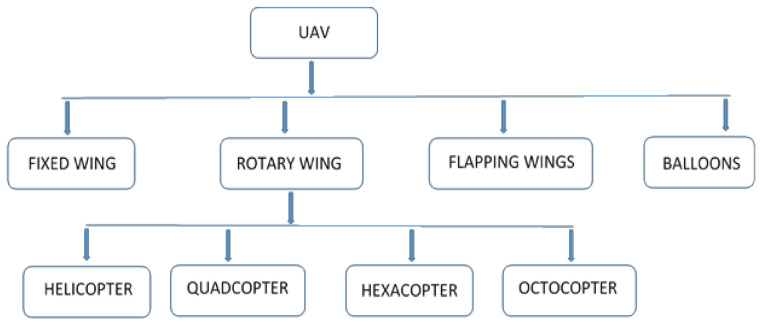
Classification of a UAV according to wing types.

Unmanned Aerial Vehicles (UAV), also known as drones, are one of the most economically important sectors of precision agriculture as they find application in weed detection, soil and field analysis, crop monitoring, nutrient deficiency detection, crop spraying, aerial planting, etc. [4]. According to Yanushevsky [5] as well as Chao, Cao, and Chen [6], an unmanned aerial vehicle can be defined as a power-driven and reusable vehicle that flies without a human pilot on-board and could be navigated using a remote control or flown autonomously with the aid of an autopilot. It finds applications in areas of surveillance, photography, rescue mission, weather monitoring, precision agriculture, payload deliveries, etc. Furthermore, it has important advantages of minimized operational cost, reduced human error, and its ability to be operated under hazardous conditions [7]. Based on its wing types in Figure 1, the UAV can be classified into the following categories.

### 1.2. Quadcopter

Hoang and Poon [8] defined a quadcopter as an aerial vehicle controlled by the rotational speed of four rotors for lift, steering, and stability. The first quadcopter, a manned vehicle though, was built in 1907, and as research progressed, there came the advent of the unmanned quadcopter by the Breguet brothers in the late 20th century [9]. The quadcopter is structured such that a brushless motor, which drives each rotor, is connected to an electronic speed controller (ESC), which in turn receives signals from the flight controller powered by a battery. The four rotors are arranged in such a way that the two diagonally opposite rotors rotate clockwise while the other two rotate in the anticlockwise direction. Each rotor produces a torque about the quadcopter’s center, and hence stability is achieved by two pairs of counter-rotating motors which give rise to a net moment/torque of zero at its center [10]. Thus, its principle of operation is such that by varying the angular velocity of one or more rotors relative to the others, the quadcopter carries out the roll, pitch, or yaw movements [11]. A typical quadcopter has the following parts [12,13,14,15]:

- Frame: It provides a physical structure, houses the electric motors and other components.
- Electric motors: They are two types of motors-brushed and brushless motors. For a quadcopter, a brushless motor is preferred due to its high thrust-to-weight ratio. The brushless motor spins the propeller, which results in lift
- Propellers: They come in different sizes and materials and are measured by diameter and pitch in the format, diameter × pitch. Pitch is a measure of how many “travels” the propeller undertakes in one revolution, while the diameter is the length of the propeller from tip to tip.
- Electronic speed controller: These are electric devices that collect PWM (Pulsating Width Modulation) signals from the flight controller and sends them to the electric motors, thus regulating their signals appropriately.
- Batteries: The battery supplies direct current (DC) power to the electric motor and all other quadcopter components via the electronic speed controllers. They come in different shapes and sizes, including alkaline, lead-acid, nickel-cadmium, lithium sulphur (Li-S), etc. Lithium polymer batteries (especially Li-S) are extensively used due to their high energy density.
- Power distribution board: It equally distributes power from the battery to the electronic speed controllers.
- Flight controller: This the brain box of the UAV as it controls the power to each electric motor based on signals received from the transmitter and onboard sensors.

## 2. Computer Vision Technology and Machine Learning

Computer vision can be defined as the branch of automation which enables computer systems to identify, see, and understand the physical world similar to the human vision. It also develops methods for the task of getting information from images. Furthermore, the application of computer vision technology and machine learning in image analysis and prediction, as regards weed, pest, and nutrient deficiency detection, has grown in recent years to meet the ever-increasing demand for fast and precise monitoring methods. When applied to drones, they are used to collect data that could be analyzed and used to provide insights to improve yield. The widely used techniques for analyzing images include machine learning (K-means, Support Vector Machine (SVM), artificial neural networks (ANN), deep learning (DL), etc.), wavelet-based filtering, vegetation indices, and regression analysis. Of all the aforementioned techniques, deep learning is gaining momentum because of its high performance and precision level [16].

### 2.1. Deep Learning Algorithm

Deep learning is a branch of machine learning comprising several processing layers that transform and learn a data representation hierarchically [1]. It is made up of the input, hidden, and output layers, with neurons in each layer connected to neurons in the corresponding layer and hence could be likened to the neuronal structure of the human brain [17]. Deep learning can be categorized into generative and discriminative models. Common examples of generative deep learning models include the autoencoders [18], deep belief networks [19], restricted Boltzmann machines [20], and generative adversarial networks [21]. Examples of discriminative deep learning models are recurrent neural networks [22], long and short-term memory [23], and convolutional neural networks (CNN) [24].

**Figure 2:**
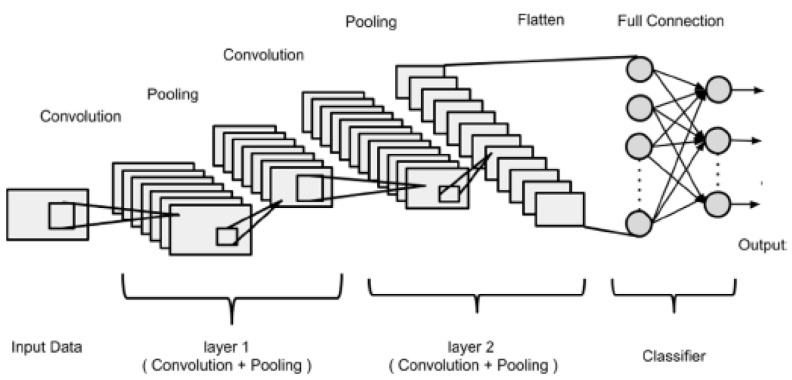
CNN architecture showing its different layers.

The CNN model is widely used in agriculture for classification, recognition, and detection tasks due to its equivalent representation, sparse interaction, and parameter sharing capabilities [25]. It consists of the input, convolutional, pooling, fully connected, and output layers. The convolutional layer consists of an array of weights that extract features (edges, contours, strokes, textures, orientation, color, etc.) from input data. These weights are used to generate feature maps when the sum of the dot product between the weights and the image values is computed. The ReLU function, which is the most commonly used activation function for CNN, is employed to add non-linearity and avoid network saturation [26]. In a bid to retain relevant information, the pooling layer (especially the max-pooling function) minimizes the spatial dimensions of the feature map and computation load in the network [27]. Thereafter, in the fully connected layer, classification occurs and a list of outputs are produced, which is narrowed down to one output when the softmax function acts on it. Similar to a traditional neural network, a CNN aims to optimize the cost function, and this is achieved by iterating the backpropagation algorithm via gradient descent to adjust the weights. CNN models can either be trained from scratch (by initializing the weights to be optimized) or through transfer learning. Transfer learning involves utilizing weights of pre-trained models, especially in a situation of deficit in training data, in order to improve model performance and prevent over-fitting. Examples of pre-trained CNN models are ResNet, VGGNet, AlexNet, MobileNet, GoogleNet, DenseNet, Inception, etc. Figure 2 shows the architecture of the CNN model adapted from ref. [23].

Deep learning algorithms are primarily theories, and hence, the implementation of these algorithms requires a hardware-software interface known as embedded systems. Embedded systems (e.g., Nvidia Jetson TX series, Raspberry Pi, Intel Edison, etc.) are defined according to ref. [28] as hardware-built systems comprising of memory chips with custom software programmed on it and their applications could be seen in driverless cars [29], monitoring systems [30], weed detection [31], etc.

### 2.2. Embedded Systems

The application of deep learning on embedded systems is a step in the ongoing technological revolution. This could be evident in the development of autonomous cars, drones, smart homes, cities, intelligent transportation, healthcare, video surveillance, etc. These embedded systems are hardware-software interfaces, and they can be used to inference deep learning models. Examples of embedded systems include Raspberry Pi, Nvidia Jetson Series, Intel Edison, Intel UP, Ordroid U3+, etc.

### 2.3. Raspberry Pi

**Figure 3:**
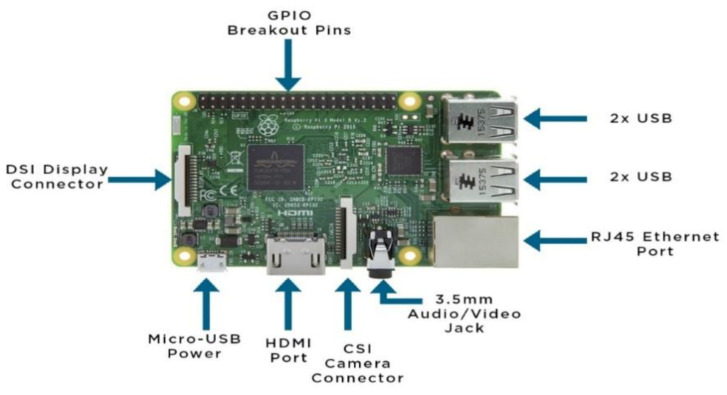
Typical Parts of a Raspberry Pi 3B Model (adapted from ref. [34]).

This is a credit-card-like single-board computer that uses the open-source Linux operating system and is used to provide access to the internet and connect automatic systems [32]. It was initially developed by the Raspberry Pi foundation in the United Kingdom to assist students in learning basic programming skills. It has evolved through several models since its first release in 2012, with significant improvements being noticed in each model released. The available models are model A, model B, model B+, model 2B, model zero, model zero W, model 3B, model 3B+, model 4B (2 GB), and model 4B (4 GB) with a typical example shown in Figure 3 [33].

### 2.4. Sprayer Module

The use of agricultural drones in spraying chemicals across farms is widespread due to the speed and effectiveness of the spraying process. The Sprayer module is a system that functions to spray agricultural inputs such as fertilizers, pesticides, and herbicides on plants. It consists of the following: nozzle—For spraying the agricultural input; tank- to store the liquid for spraying; pump—To channel the liquid from the tank to the nozzle; pressure gauge—To prevent the agricultural input from dripping; controller—Controls the speed of the pump.

## 3. Related Research Works

Alsalam et al. [35] recommended the use of a deep learning technique for more precise weed detection in their study and implemented a UAV that could change its planned path, approach a target and perform a specific action such as spraying of herbicides. Vikhram et al. [31] implemented a smartweed detection and herbicide sprayer robot that detected weeds using morphological thresholding, erosion, and dilation using a Raspberry Pi 3B, which had a sprayer module attached to conduct selective spray of herbicides. Olsen et al. [36] employed the DeepWeeds dataset to train a CNN model for weed classification on an Nvidia GTX 1080Ti GPU implemented on a ground robot. Dos Santos Ferreira et al. [37] believed that the application of herbicides yields better results if the treatment is targeted to the specific class of weeds (grass or broadleaf). Hence, they trained a convolutional neural network (CNN) on the Caffe framework to detect broadleaf and grass weeds on soybean farmland. To the best of the authors’ knowledge, research focusing on mounting a deep learning (DL) inference-embedded device on a UAV for spraying a particular herbicide on a specific class of weed has not been explored yet. This study focuses on developing a modular UAV for real-time detection of weeds and the selective spray of a particular herbicide on a specific class of weed using deep learning algorithms deployed on a Raspberry Pi 3B and mounted on a UAV. The objectives of this research work are:

- To train a CNN model through transfer learning on a pre-trained ResNet50 model.
- To deploy the trained model on a Raspberry Pi 3B and incorporate a sprayer module.
- To build a quadcopter from a readily available kit.
- To mount the Raspberry Pi together with the sprayer module on the quadcopter and test run.
- To proffer noteworthy recommendations.

## 4. Materials and Methods

### 4.1. Acquisition of Datasets

**Figure 4:**
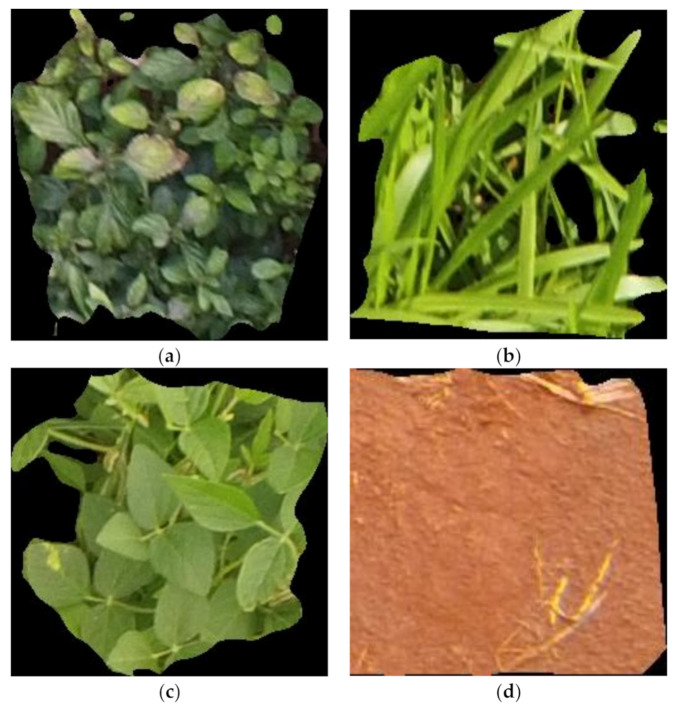
(**a**) An example of the broadleaf image dataset; (**b**) an example of the grass weed image dataset; (**c**) an example of the soybean image dataset; (**d**) an example of the soil image dataset.

The dataset used in this work is about one-third of the soybean weed dataset obtained from the public dataset source, Kaggle. It consists of soil, soybean, grass weeds, and broadleaf weeds in a total of 6109 images. The image datasets, created by Dos Santos Ferreira et al. [37], were segmented with the aid of the SLIC algorithm on the pynovisao software. A DJI Phantom 3 professional UAV, equipped with a Red, Green, Blue (RGB) camera, was used to capture the images from a height of 4 m above the ground. Figure 4a–d shows examples of the soybean weed datasets.

### 4.2. Training the CNN Model

The soybean weed dataset was divided in a ratio of 7:2:1 into training, validation, and test dataset. With an input shape of 224 × 224 × 3 and a batch size of 20, the datasets were used to train the ResNet50, through transfer learning, for 10 epochs on Google Colaboratory. The CNN model was trained on the TensorFlow framework carried out a 4 GB RAM Intel^®^ Celeron^®^ CPU 1007U @ 1.50 GHz. During compilation of the model, the Adam optimizer, categorical cross-entropy loss, and accuracy metric were employed. After the training procedure, the CNN model was converted to a TensorFlow lite format to be efficiently readable by the Raspberry Pi interpreter. Further, an optimization technique known as quantization was carried out to optimize the CNN model for both latency and size hence, improving its performance.

### 4.3. Embedded System for the Smart Herbicide Sprayer

**Figure 5:**
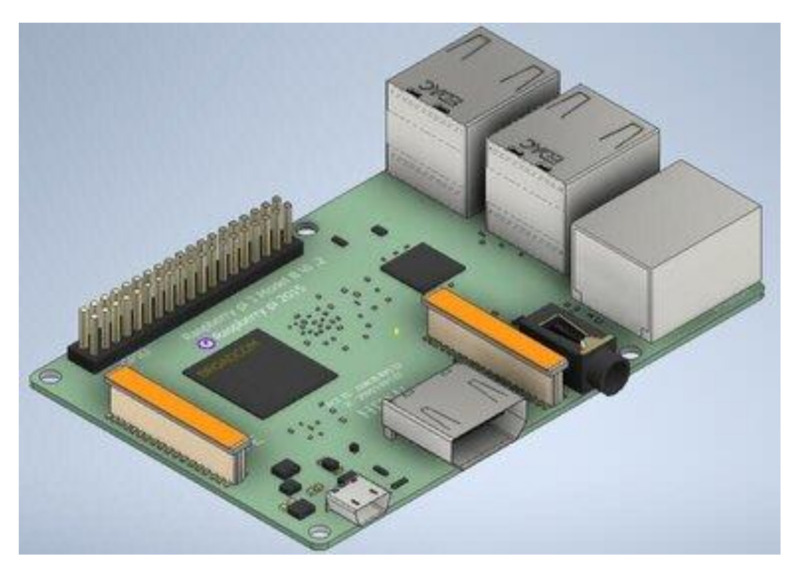
The layout of the Raspberry Pi 3B.

The embedded system used is the Raspberry Pi 3B. It consists of the quadcore 64-bit ARM cortex @ 1.2 GHz, 1 GB RAM, 4 USB 2.0 ports, HDMI ports, camera serial interface, display serial interface, micro-USB power port 40 GPIO pins, and SD card slot. The raspbian is the operating system recommended and hence being employed for use on the Raspberry Pi 3B. Figure 5 provides a pictorial representation of the Raspberry Pi 3B.

### 4.4. Setup for the Raspberry Pi for DL Applications

The components required for the setup are: A 2.5A 5.1 V micro-USB power supply, an SD card ranging from 16 to 32 GB, keyboard and mouse, an HDMI cable, a Pi camera or USB camera, a computer or television screen. The New out of Box (NOOBS) installation manager is the easiest way of installing the raspbian operating system on the SD card using a personal computer (PC) and an SD card reader. The raspbian operating system was installed using NOOBS. Moreover, the Tensorflow and OpenCV libraries were installed in a virtual environment created on the Raspberry Pi. The trained model from the PC was transferred to the Raspberry Pi using the FileZilla client software.

### 4.5. Materials for the Smart Herbicide Sprayer

The required materials for the system work include Quadcopter kit, ZOP power 5500 MAH 11.1 V li-poly battery, Balance smart battery charger, Raspberry Pi 3B kit, Raspberry Pi camera V2, CMU Flysky FS-I6 6 channel transmitter/receiver set, single/1 channel 5VDC 10 A relay module development board, BMT mini submersible water pump, Anker PowerCore 13,000 MAH power bank, universal tail landing gear skid for DJI F450, pressure washer spray nozzle, Pixnor APM 2.6 MWC GPS compass antenna folding fixed mount bracket, Mirthhobby RC anti-vibration plate, battery holder 8× AA with black leads, male battery connector plug, 1.5 V alkaline AA size battery (24 pieces/pack). Calculations were carried out to allow for component compatibility as the quadcopter kit would be purchased with an emphasis on its weight and power specifications.

### 4.6. Calculation for Component Compatibility

#### 4.6.1. ESC Calculation

The amperage rating of the ESC should be 20–50% more than the amperage rating of the electric motor.

(1) min ESC amperage= 1.2 × min amp rating of a motor

(2) max ESC amperage = 1.5 × max amp rating of a motor

#### 4.6.2. Battery Calculation

(3) Disch. current = battery capacity × C rating,

The higher the rate of current discharge rate, the higher the capacity of the battery to withstand overheating. Moreover, the ESC amperage rating should not exceed the battery discharge current.

(4) max current drawn by the motors = no. of motors × max current drawn by 1 motor,

#### 4.6.3. Thrust Calculation

For a quadcopter to be able to lift off the ground, the total thrust to total weight ratio should be at least 2:1.

(5) $$Thrust\;provided\;by\;the\;propellers\;=\;\frac{2}{4}\times \;total\;weight\;of\;quadcopter$$

(6) $$Thrust\;provided\;by\;the\;propellers\;=\;\frac{\pi \;\times \;D^{2}\;\times \;v\;\times \;\Delta v\;\times \;p}{4}$$

Where D = diameter of propeller

v = velocity of air

Δ = velocity of accelerated air

With the assumption that it equals 11.61 m/s at 78% ideal efficiency

*p* = density of air

The result of the calculation is presented in Table 1.

From the specification of the purchased components, the electric motors draw a maximum current of 80 A, which is less than the battery discharge current. Moreover, the ESCs are compatible with the electric motor since it has a current rating of 30 A, which is higher than that of the electric motor (12 A). Furthermore, the ESC is designed to handle batteries ranging from two to three cells, and the battery being used in this work has three cells. From the thrust calculations, the thrust of each motor obtained is seen to be more than 2/4 of the weight of the quadcopter hence lift is assured. The frame chosen is made up of carbon fiber which is known for its lightweight and low inertia.

### 4.7. Assembling the Smart Herbicide Sprayer

**Figure 6:**
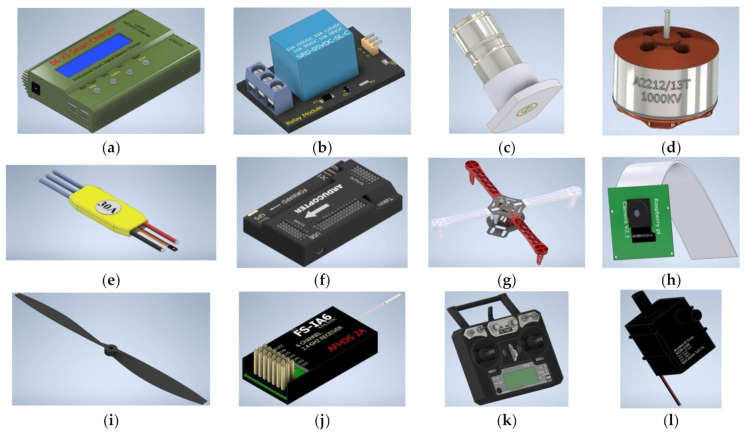
(**a**) Battery Charger (**b**) Relay module (**c**) Spray Nozzle (**d**) Brushless Electronic Motor (**e**) Electronic Speed Controller (**f**) Flight controller (**g**) Frame (**h**) Pi-Camera (**i**) Propeller (**j**) Radio receiver (**k**) Radio Transmitter (**l**) Spray Pump.

The following tools were required to assemble the smart herbicide sprayer: soldering iron and soldering flux, screwdriver, component vice for keeping the components in place while working, wire strippers, screws, heat gun/lighter, scissors, needle-nose plier, and multimeter. Table 2 gives the specifications of some components of the smart herbicide sprayer, Figure 6 shows a pictorial representation of these components, while Table 3 displays the cost analysis.

#### 4.7.1. Procedures for Assembling the Smart Herbicide Sprayer

The procedures are detailed: Solder the ESCs to the bottom power distribution board. The red wire of the ESC should be soldered on the positive (+) contact, while the black wire should be soldered on the negative (−) contact. Similarly, wires of the battery should be soldered on the power distribution board; fix the four arms of the frame and landing gears to the bottom power distribution board with screws. Thereafter, the motors should be mounted on the frame using screws. It should be ensured that the screws do not touch the copper wires in the motor; solder the male bullet plugs on each of the three wires of the brushless motors and female bullet plugs on each of the three wires of the ESCs. Then put a heat shrink tube on the bullet plugs and shrink the tube using a lighter or heat gun; mount the top board onto the frame using screws. Then fix the APM 2.8 flight controller, already placed in a vibration absorber plate, onto the top board of the frame. The M8N GPS should also be mounted on the GPS holder placed alongside the flight controller; connect the ESC cable set to channels 1 to 4 at the input section of the flight controller.

Further, connect the 3-wired cable set from channels 1 to 4 on the receiver to channels 1 to 4 at the output region of the flight controller. Thereafter, connect the GPS to the flight controller; connect the ESCs to the motors and then calibrate the accelerometer, radio, GPS, and transmitter by connecting the flight controller to a PC with the mission planner software already installed; calibrate the ESCs by connecting each ESC to the throttle channel (channel 3) of the receiver and also connecting it to the battery. Then using the transmitter, take the joystick to full throttle and wait till the ESC makes a beep sound before disconnecting; connect all components of the quadcopter and check if all motors are rotating in the desired direction. Ideally, two motors should rotate in the clockwise direction while the other two opposite motors should rotate in the anticlockwise direction. If they are not moving as expected, swap any of the two wires of the ESC connected to the motors; fix the propellers on the shaft of the motor using the motor accessories and connect the battery fastened onto the frame with the battery straps; connect the GPIO 17 and 18 of the Raspberry Pi, each to the signal contacts of the relays by soldering.

**Figure 7:**
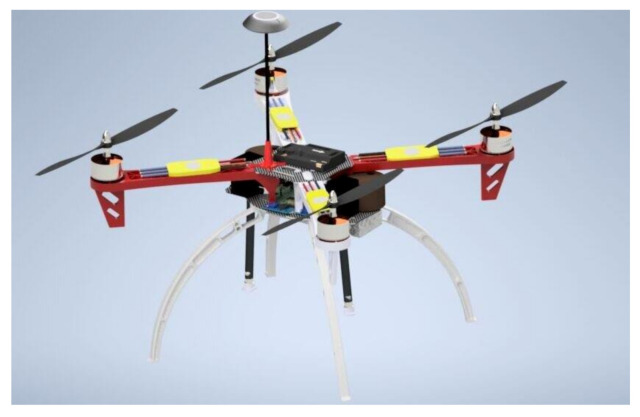
A design of the smart herbicide sprayer.

**Figure 8:**
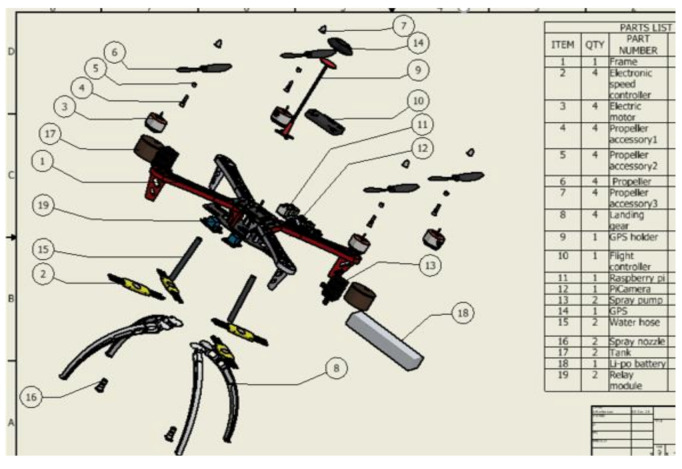
Exploded view and part list of the UAV prototype.

Further, connect pin 2 and pin 4 each to the VCC contacts of the relays. Pin 6 and pin 34 should be connected to the ground contact of each relay. Then connect the positive wire of the spray pumps to the ‘normally open’ contacts of each relay and connect the 12 V DC power source to the common of the relay. The ground or negative end of both the 12 V power source and spray pump should be connected; ensure that the picamera is well placed on the CSI port of the Raspberry Pi. Moreover, connect an 8 mm water hose from the spray pump to the tank, fasten the Raspberry Pi, relay, spray pumps, and tanks on the assembled quadcopter. Figure 7 presents the setup design and Figure 8 presents the exploded diagram of the setup.

### 4.8. Principle of Operation

Before any flight operation, the following checks are to be conducted: inspection of the frame for any physical damage or crack; inspection of the motors and propellers for damage or presence of debris and ensure that both are secured correctly; inspect all electrical components for correct functionality; inspection of the installation of parts that are removable such as the battery. The smartweed detector would operate so that pictures taken by the picamera, connected to the Raspberry Pi, which is mounted on the quadcopter, would be analyzed by the deployed CNN model. When a broadleaf weed is detected, the GPIO 18 is activated to supply 3.3 V for 3 s. This voltage (from GPIO 18) and the 5 V voltage common collector (VCC) would be supplied to a DC relay module. When the signal voltage from GPIO 18 is supplied to the relay, the relay closes the circuit to connect the spray pump 1 with 12 V DC power. The spray pump in turn draws a broadleaf weed herbicide from tank 1 and pumps it through the nozzle.

**Figure 9:**
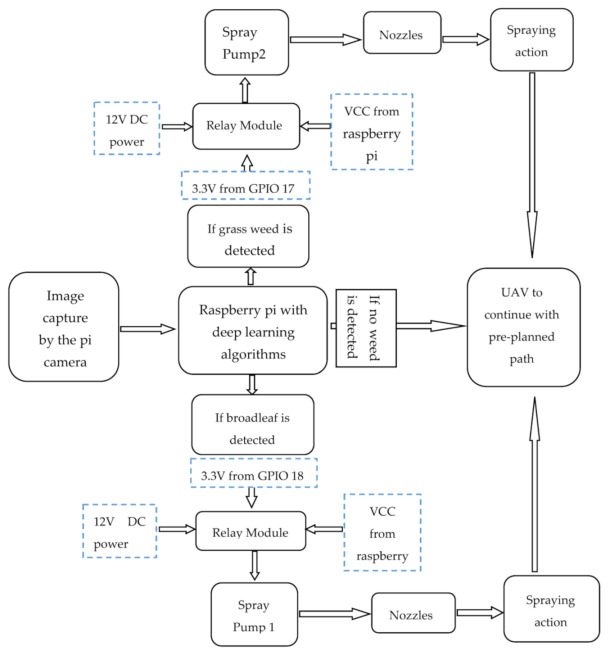
Schematic diagram explaining the working principle of the smart herbicide sprayer.

On the other hand, when a grass weed is detected, the GPIO 17 is activated to supply 3.3 V for 3 s. This voltage is also combined with 5 V from the Raspberry Pi’s VCC to turn on a relay, which connects spray pump 2 to a 12 V DC power. Spray pump 2 then draws grass weed herbicide from tank2 and pumps it through the nozzle. However, when a soybean plant is detected, no GPIO pin is activated, and hence no spraying action occurs. Figure 9 shows a block diagram for the principle of operation.

## 5. Results

### 5.1. Outcome of the Model Training Procedure

**Figure 10:**
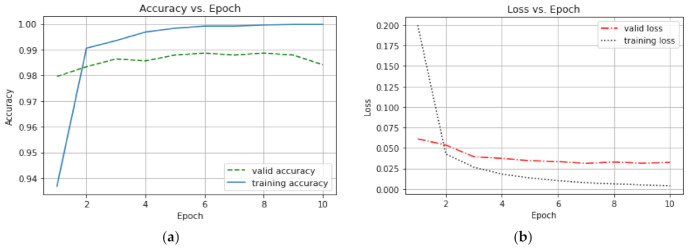
(**a**) A graph of accuracy vs epoch; (**b**) A graph of loss vs epoch.

The CNN model generated 8196 trainable parameters from training 6109 images of input shape 224 × 224 × 3. After the training procedure, training and validation accuracies of 99.98% and 98.4%, respectively, were obtained. Further, training and validation losses of 0.0039 and 0.0323 were equally obtained. Figure 10 shows the accuracy and loss graphs of the CNN model.

According to the accuracy versus epoch and loss versus epoch graphs above, it was noticed that the best accuracy was achieved at the ninth epoch. A low variance error, which is the difference between the training and validation accuracies, of 1.1% was recorded; hence there was no over-fitting of the training data. Further, the training and validation losses were below 2.5% and 5%, respectively, depicting that the model learned the features perfectly well.

### 5.2. Outcome of Incorporating the Sprayer Module with the Raspberry Pi

**Figure 11:**
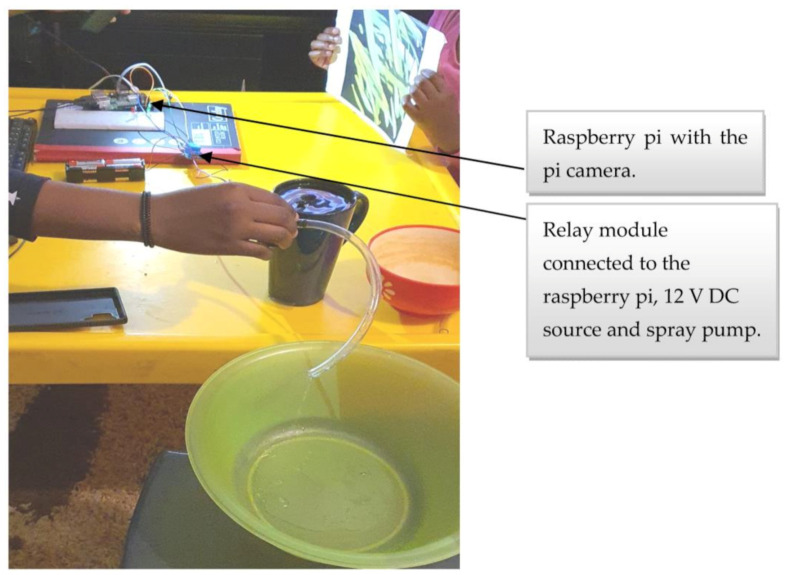
Testing the sprayer module.

The Raspberry Pi 3 with the CNN model already deployed was linked with the sprayer module and tested for spraying. The positive end of the spray pump was connected to ‘normally open’ and a 12 V DC power source connected to the common of the relay. Further, when broadleaf weed was detected, GPIO 18 was activated to supply 3.3 V together with the VCC from the Raspberry Pi, which served as inputs to the relay to turn on spray pump 1. Similarly, when grass weed was detected, GPIO17 was activated to supply 3.3 V together with the VCC from the Raspberry Pi, which served as inputs to the relay to turn on spray pump 2. The sprayer module was tested using an image of the grass weed, as shown in Figure 11 and the spray pump turned on when the grass weed image was brought close to the pi camera.

### 5.3. Assembling the Quadcopter Kit

**Figure 12:**
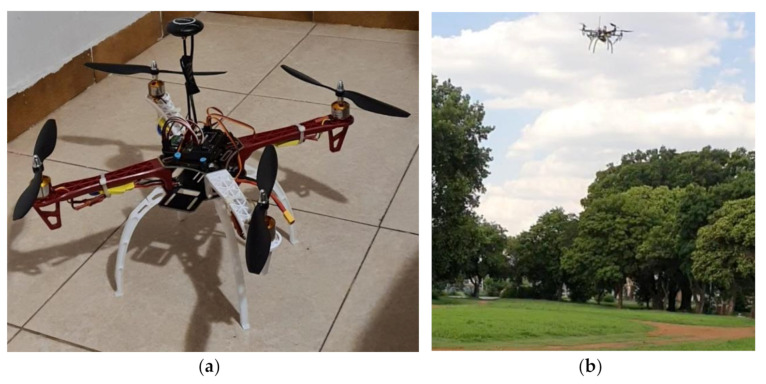
(**a**) Assembled quadcopter; (**b**) First test-run of the quadcopter.

The procedures for assembling a quadcopter kit, as explained in Section 4.7.1, were strictly followed during the building of the quadcopter. Figure 12a shows the assembled quadcopter. The quadcopter was first tested and was found to be in good condition with a time of flight of about 15 min. Figure 12b shows the quadcopter during its first test-run.

### 5.4. Test-Running the Smartweed Detector and Selective Herbicide Sprayer

**Figure 13:**
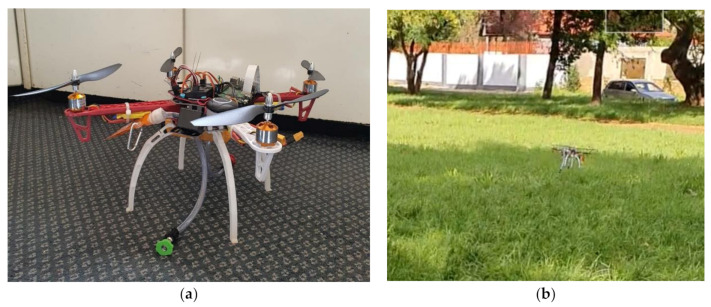
(**a**) Quadcopter with the sprayer module incorporated; (**b**) Test-running the smartweed detector and selective herbicide sprayer.

The sprayer module, and the Raspberry Pi were incorporated on the assembled quadcopter to form the smartweed detector and selective herbicide sprayer, as shown in Figure 13a. A final test-run was carried out on the smart herbicide sprayer, and it was observed that it could detect broadleaf weed or grass weed accurately in less than a second at the height of 50 cm above the ground and spray herbicides effectively. However, spraying of herbicides occurred after two weed detection attempts. This means that the sprayer must detect a weed twice consecutively before spraying the appropriate herbicide. Although the smart sprayer commences spraying operation immediately after a grass weed is detected, it sprays for 10 s instead of the envisaged 3 s. Figure 13b displays the smart selective herbicide sprayer during the test-running operation.

### 5.5. Discussion

Recent research studies focused on DL models [38,39] involve just the training of deep learning models for weed detection. However, this study focuses not only on training the DL model but also on the implementation process via the deployment of DL on a UAV that could be used to access hard-to-reach areas. Moreover, in other studies [31,35], ground robots and UAVs have been proposed for the detection of weeds using other techniques such as morphological thresholding, dilation, etc. but DL employed in this study provides better detection accuracy than those of previous studies.

Furthermore, in the literature on unmanned systems and remote sensing [36], the herbicide sprayers are designed to spray the same herbicide, regardless of the type of weed, and this has been proven to be less effective in eliminating weeds. Hence, a selective herbicide sprayer was developed in this study whereby different herbicides are sprayed on grass and broadleaf weeds. This is envisaged to improve crop yield leading to a corresponding decrease in production cost.

Although the contributions mentioned above have been achieved in this study, there were a few highlighted variations from what was anticipated. This has to do with the time interval for spraying the herbicides and spraying the appropriate herbicide at the second attempt when there is an immediate change in weed type detected. The latter is due to a ‘clean-up’ issue on the Raspberry Pi as it correctly detects the right weed but fails to activate the appropriate GPIO pin at the first attempt accurately. Nevertheless, the results obtained from this research create the opportunity for more research to be conducted in this field of precision agriculture for further improvements.

## 6. Conclusions and Recommendations

### 6.1. Conclusions

This research work provides details on the development of a smartweed detector and selective herbicide sprayer that detects and sprays weeds on farmland. This was achieved by deploying deep learning algorithms on an embedded system mounted on a quadcopter. As a step in the right direction in the domain of precision agriculture, it envisages solving the problem of poor harvest due to competition for nutrients by weeds and effective elimination of weeds through its selective spraying ability. This research has addressed the following:

- A CNN model was trained through transfer learning on the soybean dataset. The CNN model was converted to TensorFlow lite format and deployed on the Raspberry Pi 3. Training and validation accuracies of 99.98% and 98.4% were obtained respectively with an insignificant variance error. Moreover, training and validation losses of 0.0039 and 0.0323 respectively reveal that the proposed model was appropriate.
- A quadcopter was built, and its components were assembled with light-weight and strong materials to improve its efficiency. It consists of the electric motor, ESC, propellers, frame, li-po battery, flight controller, GPS, receiver. In this research work, a quadcopter was built and was able to perform as pre-planned.
- A sprayer module which consists of a relay, Raspberry Pi 3, spray pump, 12 V DC source, water hose, and the tank, was built. It operated in such a way that when a weed was detected based on the deep learning algorithms deployed on the Raspberry Pi, GPIO 17 or GPIO 18 were activated to supply 3.3 V, which turned on a DC relay to spray herbicides accordingly.
- The sprayer module was mounted on the quadcopter, and from the test-running operation carried out, broadleaf and grass weeds were accurately detected and spraying of herbicides according to the weed type occurred in less than a second.

### 6.2. Recommendations for Future Research

In a bid to establish a basis on which improvements could be made in this research work, the following recommendations have been made by authors: the use of an RGB camera with a higher resolution quality would enable a better picture capture from a higher height above the ground and hence a more accurate detection at higher heights; employing the use of DL hardware accelerators such as the Intel Movidius compute stick would lead to a reduced inference time which is an important criterion for real-time detection; implementing DL algorithms on better and more efficient embedded systems such as the Nvidia Jetson TX2, FPGA, etc. would improve the spraying actuation challenge experienced with the Raspberry Pi in this work; the field of DL is broad and evolving with swift advancement in technology, hence extensive research on the use of better DL detection algorithms, such as YOLO, is imperative; powering the smart herbicide sprayer with a renewable energy source such as solar would increase the flight time as the estimated time of flight with batteries is about 15–20 min; making the smart herbicide sprayer autonomous by planning its flight path using a grand control station.

## Figures and Tables

**Table 1:** Calculations performed to ascertain the UAV’s component compatibility.

Parameters	Value Obtained
Minimum amperage	14.4 A
Maximum amperage	18 A
Discharge current	247.5 A
Maximum current drawn by the motors	80 A
Thrust provided by the propellers	340.47 N

**Table 2:** Specification of selected smart herbicide sprayer components/accessories.

S/N	Name of Component	Specification
1	XXD A2212 brushless outrunner electric motor	kV: 1000 Current rating: 12 A/60 s No-load Current: 10 V: 0.5 A Number of cells: 2–3 lithium polymer battery Dimensions: Ø27.5 × 30 mm
2	Electronic speed controller	Continuous current: 30 A Burst current: 40 A Input voltage: 2–3 cell lithium-polymer battery BEC: 2 A/5 V (linear mode) Size: 45 mm (L) × 24 mm (W) × 11 mm (H); Weight: 25 g
3	APM 2.8 flight controller	Made up of a 3-axis gyro, accelerometer, and barometer. 4-megabyte dataflash chip. Optional off-board GPS LEA-6CH module with a compass.
4	Propeller	Diameter: 10” Pitch: 4.5”
5	Flysky FS-I6 CH transmitter/receiver set	Transmitter: Channel: 6 Modulation type: GPSK RF range: 2.408–2.475 GHz Bandwidth: 500 kHz Receiver: FS-IAS 6-channel
6	BMT mini subm. water pump	Working voltage: 12 V DC Working current: 400 mA Max flow: 240 L/HPump life: >30,000 h Inlet diameter: 8 mm Outlet diameter: 8 mm
7	ZOP power 11.1 V 3S lithium-polymer battery	Capacity: 5500 MAH Continuous discharge rate: 45 C Size: 40 × 46.5 × 138
8	B6 V3 Smart Balance Charger	DC Input voltage: 11–18 v Charge power: 80 W Discharge power: 10 W Charge current range: 0.1–6 A Discharge current range: 0.1–2 A
9	Single/1 channel 5VDC 10 A relay module development board	Control Voltage: 5 V DC Max Control Capacity: 10 A@250 VAC or 10 A@30 VDC Size: 41 × 16 × 16 mm Weight: 12 g

**Table 3:** Cost Analysis of the smart herbicide sprayer components.

S/N	Components	Cost (Rands)
1	Quadcopter Kit	3120
2	ZOP power 5500 MAH 11.1 V li-poly battery	2250
3	Balance smart battery charger	675
4	Raspberry Pi 3B kit	1400
5	Raspberry Pi camera V2	582
6	CMU Flysky FS-I6 6 channel transmitter/receiver set	1451
7	Single/1 channel 5 VDC 10 A relay module development board	61
8	BMT mini subm. water pump	153
9	Anker PowerCore 13,000 MAH power bank	805
10	Universal tail landing gear skid for DJI F450	236
11	Pressure washer spray nozzle	311
12	Pixnor APM 2.6 MWC GPS compass antenna folding fixed mount bracket	214
13	Mirthhobby RC anti-vibration plate	328
14	Battery holder 8× AA with leads black	21
15	Battery connector male plug	19
16	AA size batter 1.5 V alkaline 24 pieces/pack	120
	Total	R11 746/790 USD

## Data Availability

The study didn’t report any data.
